# Gadolinium – Determination of gadolinium and its compounds in workplace air using inductively coupled plasma mass spectrometry (ICP-MS): Air Monitoring Method – Translation of the German version from 2024

**DOI:** 10.34865/am744054e10_1or

**Published:** 2025-03-31

**Authors:** Cornelia Wippich, Katrin Pitzke, Thomas Göen, Ralph Hebisch, Uta Lewin-Kretzschmar, Andrea Hartwig, MAK Commission

**Affiliations:** 1 Institute for Occupational Safety and Health of the DGUV (IFA). German Social Accident Insurance (DGUV) Alte Heerstraße 111 53757 Sankt Augustin Germany; 2 Friedrich-Alexander-Universität Erlangen-Nürnberg. Institute and Outpatient Clinic of Occupational, Social, and Environmental Medicine Henkestraße 9–11 91054 Erlangen Germany; 3 Federal Institute for Occupational Safety and Health (BAuA) Friedrich-Henkel-Weg 1–25 44139 Dortmund Germany; 4 German Social Accident Insurance, Institution for the raw materials and chemical industry, Prevention - Department of Hazardous Substances, Biological Agents and Analytical Chemistry Kurfürsten-Anlage 62 69115 Heidelberg Germany; 5 Institute of Applied Biosciences. Department of Food Chemistry and Toxicology. Karlsruhe Institute of Technology (KIT) Adenauerring 20a, Building 50.41 76131 Karlsruhe Germany; 6 Permanent Senate Commission for the Investigation of Health Hazards of Chemical Compounds in the Work Area. Deutsche Forschungsgemeinschaft, Kennedyallee 40, 53175 Bonn, Germany. Further information: Permanent Senate Commission for the Investigation of Health Hazards of Chemical Compounds in the Work Area | DFG

**Keywords:** gadolinium, air analyses, analytical method, workplace measurement, hazardous substance, mass spectrometry with inductively coupled plasma, ICP-MS, membrane filter, acid digestion

## Abstract

The working group “Air Analyses” of the German Senate Commission for the Investigation of Health Hazards of Chemical Compounds in the Work Area (MAK Commission) developed and verified the presented analytical method. This analytical method is a validated measurement procedure for the determination of gadolinium [7440-54-2] and its compounds in workplace air in a concentration range of one tenth up to twice of the general dust limit value for the respirable dust fraction in Germany of 1.25 mg/m^3^. For sampling, a defined volume of air is drawn through a membrane filter (cellulose nitrate). The flow rate is set to 10 l/min and sampling duration is 2 hours. Gadolinium is extracted with a mixture of hydrochloric and nitric acid or just nitric acid, depending on the digesting method – open hot-block or microwave-assisted pressure digestion. The samples are subsequently analysed using mass spectrometry with inductively coupled plasma (ICP-MS). The quantitative determination is based on a calibration function. The limit of quantification is 0.002 µg/m^3^ (open hot-block digestion) and 0.001 µg/m^3^ (microwave-assisted pressure digestion), respectively, based on an air sample volume of 1200 l and 20 ml digestion volume. The mean recovery is 95.1% (open hot-block digestion) and 94.7% (microwave-assisted pressure digestion), respectively. The expanded uncertainty for the validation range of 0.167 to 2.5 mg/m^3^ is 23.5 to 25.0%.

**Table:** 

**Method number**	1
**Application**	Air analysis
**Analytical principle**	Inductively coupled plasma mass spectrometry (ICP-MS)

## 1 Characteristics of the method

**Table:** 

**Precision:**	Coefficient of variation:	*V_x_* = 4.0 to 4.5%
**Reproducibility:**	Coefficient of variation:	*V_x_* = 0.70 to 2.8%
	Expanded uncertainty:	*U* = 23.5 to 25%
	in the range from 0.167 to 2.5 mg/m^3^ and n = 8	
**Limit of quantification:**	Open hot-block digestion:	0.013 µg/l
	Microwave-assisted pressure digestion:	0.005 µg/l
	0.002 and 0.001 µg/m^3^, respectively, for an air sample volume of 1200 l and a sampling period of 2 h	
**Recovery:**	*η* = 94.4–96.4%
**Sampling recommendation:**	Sampling period:	2 h
	Air sample volume:	1200 l
	Volumetric flow rate:	10 l/min
	For short-term measurements:	15 min; 10 l/min

## 2 Description of the substance

### Gadolinium [7440-54-2]

Gadolinium is a light grey, ductile and flammable metal that has the element symbol Gd (relative atomic mass 157.25 u, melting point 1313 °C, boiling point 3273 °C, density 7.89 g/cm^3^).

Gadolinium is a rare-earth metal that belongs to the lanthanide series. To extract gadolinium, the ore in which it occurs is first digested with a sodium hydroxide solution at high temperature. Filtration and the precipitation of thorium in the form of its hydroxide are followed by several consecutive liquid-liquid extraction steps to separate the rare-earth metals from one another. This produces gadolinium salts, which are then converted to oxides by annealing. The oxides are converted to gadolinium fluoride and gadolinium chloride by reacting with hydrogen fluoride and hydrochloric acid, respectively, and then reduced to the element gadolinium in a metallothermic reduction reaction using mainly calcium as a reducing agent. A final purification step is carried out by vacuum distillation (Bünzli and Mcgill 2018). Gadolinium is used for many different applications, for example as a component of magnetic storage devices and for the manufacture of optical waveguides and optical storage media. Gadolinium oxide is used as an absorber in nuclear fuel (Bünzli and Mcgill 2018). For several decades, gadolinium has been used as a component of contrast agents for imaging procedures such as magnetic resonance imaging (MRI). However, patients have been found to have deposits of the element gadolinium, which are suspected of causing adverse health effects (Ramalho et al. 2016).

Assessment criteria have not yet been established for gadolinium and a MAK value has not been derived. The method for determining gadolinium and its compounds in the air was validated using the general limit value for dust, respirable fraction (R dust), of 1.25 mg/m^3^ (AGS 2024).

## 3 General principles

This analytical method is used to concurrently determine the levels of gadolinium and of gadolinium compounds in the workplace air in a concentration range from one tenth to twice the currently valid general limit value for dust of 1.25 mg/m^3^ R (AGS 2024).

Samples are taken by drawing a defined volume of air through a membrane filter (nitrocellulose) by means of a suitable sampling pump. After acid digestion, the amount of gadolinium deposited on the filter is determined by inductively coupled plasma mass spectrometry. The quantitative evaluation is based on the calibration curve obtained by plotting the quotients of the gadolinium concentrations in the calibration standards and the lutetium internal standard against the intensities of the measured pulses.

## 4 Equipment, chemicals and solutions

### 4.1 Equipment

For sampling:

- Sampling pump for personal and stationary sampling (maintains adequate performance in spite of the drop in pressure after inserting the carriers), suitable for a flow rate of 10 l/min in conformance with DIN EN ISO 13137 (DIN 2023 b) (e.g. type SG10-2, from GSA Messgerätebau GmbH, 40880 Ratingen, Germany)
- FSP 10 sampling head for the respirable dust fraction with cyclone separator (e.g. from GSA Messgerätebau GmbH, 40880 Ratingen, Germany)
- PGP filter cassette for the personal sampling system for hazardous substances, plastic, with covers for 37-mm filters (e.g. from GSA Messgerätebau GmbH, 40880 Ratingen, Germany)
- Membrane filters, Ø 37 mm, pore size 8.0 µm, nitrocellulose. Each batch analysed for metal content (e.g. nitrocellulose membrane filters, from Sartorius AG, 37079 Göttingen, Germany (or filters of equivalent quality))
- Supporting sieve, 37 mm (e.g. from Metaq GmbH, 42115 Wuppertal, Germany)
- Gas meter or flow meter (e.g. TSI flowmeter 4146, from TSI GmbH, 52068 Aachen, Germany)

For sample preparation:

- Heating block thermostat made of metal or graphite with external time/temperature control (e.g. from Gebr. Liebisch GmbH & Co, 33649 Bielefeld, Germany)
- Digestion vessels, preferably made of quartz glass or of equivalent quality, that conform with the requirements for reaction vessels of the standard DIN 12353 (DIN 1981), graduated: diameter 19 mm, max. volume 25 ml; with standard ground-glass joints (ST 19/26), graduated in increments of 0.2 ml at least from 15 to 25 ml (e.g. from Merck Eurolab GmbH, 53797 Lohmar, Germany) diameter 23 mm, max. volume 50 ml; with standard ground-glass joints (ST 19/26), graduated in increments of 0.5 ml at least from 35 to 50 ml (e.g. from Merck Eurolab GmbH, 53797 Lohmar, Germany)
- Air cooler, preferably made of quartz glass or of equivalent quality, that conforms with the requirements of the standards DIN 12353 (DIN 1981) and DIN 12242 (DIN 2023 a), with standard inner and outer ground-glass joints (ST 19/26) for mounting onto digestion vessels (e.g. from Merck Eurolab GmbH, 53797 Lohmar, Germany)
- Glass rods, preferably made of quartz glass, with replaceable endpieces, e.g. polytetrafluoroethylene (PTFE) tubing (e.g. from Merck Eurolab GmbH, 53797 Lohmar, Germany)
- Device for microwave-assisted pressure digestion with temperature and pressure controls (e.g. ultraCLAVE, from MLS Mikrowellen-Labor-Systeme GmbH, 88299 Leutkirch im Allgäu, Germany)
- Sample vials with loose-fitting caps made of PTFE and/or quartz glass for microwave-assisted pressure digestion (e.g. from MLS Mikrowellen-Labor-Systeme GmbH, 88299 Leutkirch im Allgäu, Germany)
- PTFE racks for the sample vials used with the microwave-assisted pressure digestion system (e.g. holding up to 40 sample vials) (e.g. from MLS Mikrowellen-Labor-Systeme GmbH, 88299 Leutkirch im Allgäu, Germany)
- 5-l ultrapure water container made of perfluoroalkoxy alkane (PFA) with PTFE dispenser
- 500-ml container made of PFA with PTFE dispenser
- 500-ml spray bottle made of PFA with closure made of polypropylene (PP)
- Graduated cylinders made of PFA, maximum nominal volumes 50, 100 and 500 ml
- Caps made of polyethylene (PE) for the digestion vessels listed above (ST 19/26) (e.g. from Pöppelmann GmbH & Co KG, 49393 Lohne, Germany)
- Ceramic tweezers
- Electronic precision balance suitable for weighing samples in the range from 3 to 10 mg

For the analytical determination:

- Quadrupole ICP mass spectrometer with a collision/reaction cell and an autosampler (e.g. ICP-MS NexION 2000B, from PerkinElmer LAS GmbH, 63110 Rodgau, Germany)
- Cooled cyclonic spray chamber with inner tube made of quartz glass (e.g. from PerkinElmer LAS GmbH, 63110 Rodgau, Germany)
- Concentric quartz nebuliser, flow rate 400 µl/min (e.g. MicroMist, from PerkinElmer LAS GmbH, 63110 Rodgau, Germany)
- Containers to hold standard and calibration solutions and samples for ICP-MS: PP vials with screw caps (tested for blank values), graduated, 0.5-ml increments, maximum volume 15 ml, for autosamplers (e.g. from Greiner AG, Kremsmünster, Austria) containers, e.g. made of PP, for storing the rinsing solution for the autosampler (4 l) containers, e.g. made of PFA, for storing the dilution solution (2 l)
- Positive displacement dispenser system for precise and automated sequential dosing of internal standard and for bringing to volume with dilution solution (e.g. Microlab 600, from Hamilton Bonaduz AG, Bonaduz, Switzerland)
- Various adjustable piston pipettes to cover a range of volumes from 10 µl to 5 ml, air displacement for aqueous solutions and suspensions at a density and viscosity similar to water (e.g. from Socorex Isba S.A., Ecublens, Switzerland)
- Ultrapure water system with reverse osmosis unit for the preparation of ultrapure water (ρ ≥ 18.2 MΩ × cm at 25 °C), for the reduction of the overall metal content, and particularly for the production of water that is low in boron and alkalis (e.g. Milli-Q, from Merck KGaA, 64293 Darmstadt, Germany)

### 4.2 Chemicals

- Nitric acid, 65%, low metal content, batch certification by the manufacturer (e.g. Suprapur, from Merck KGaA, 64293 Darmstadt, Germany)
- Nitric acid, approx. 67–69%, low metal content, batch certification by the manufacturer (e.g. J.T.Baker INSTRA-ANALYZED Plus for the trace analysis of metals, from Fisher Scientific GmbH, 58239 Schwerte, Germany)
- Hydrochloric acid, 30%, low metal content, batch certification by the manufacturer (e.g. Suprapur, from Merck KGaA, 64293 Darmstadt, Germany)
- Lutetium standard for ICP, 1000 mg/l, Lu_2_O_3_ in 2–3% HNO_3_ (e.g. Certipur, traceable to NIST SRMs, from Merck KGaA, 64293, Darmstadt, Germany)
- Scandium standard for ICP, 1000 mg/l, Sc_2_O_3_ in 7% HNO_3_ (e.g. Certipur, traceable to NIST SRMs, from Merck KGaA, 64293, Darmstadt, Germany) *not required, see Note*
- Tellurium standard for ICP, 1000 mg/l, Te in 5% HNO_3_ (e.g. Specpure, traceable to NIST SRMs, Alfa Aesar, from Fisher Scientific GmbH, 58239 Schwerte, Germany) *not required, see Note*
- Yttrium standard for ICP, 1000 mg/l, Y_2_O_3_ in 5% HNO_3_ (e.g. Specpure, traceable to NIST SRMs, Alfa Aesar, from Fisher Scientific GmbH, 58239 Schwerte, Germany) *not required, see Note*
- Gadolinium standard for ICP, 10 000 mg/l, in 2–5% HNO_3_/tr. HF, (e.g. ARISTAR, traceable to NIST SRMs, from VWR International GmbH, 64295 Darmstadt, Germany)
- Gadolinium standard for ICP, 10 mg/l (2 batches: 1st for control standards and 2nd for calibration), in 2% HNO_3_, (e.g. ARISTAR, traceable to NIST SRMs, from VWR International GmbH, 64295 Darmstadt, Germany)
- Gadolinium, plasma standard for ICP, 1000 mg/l in 5% HNO_3_, (e.g. Specpure, Alfa Aesar, from Thermo Fisher Scientific GmbH, 76057 Karlsruhe, Germany)
- Gadolinium powder (≤ 40 mesh), ≥ 99.9%, rare earth oxide basis (REO) (e.g. from abcr GmbH, 76187 Karlsruhe, Germany)
- Gadolinium(III) chloride powder, anhydrous, ≥ 99.99%, REO (e.g. Apollo Scientific Ltd, Bredbury, UK)
- Au, Hf, Ir, Pd, Pt, Rh, Ru, Sb, Sn and Te multi-element standard (MES 4), 10 µg/l, in H_2_O, 10% HCl/1% HNO_3_ per 125 ml (e.g. TruQ ms Multi-Element Calibration Standard 4, from PerkinElmer LAS GmbH, 63110 Rodgau, Germany, Art. No.: N9300234)
- B, Ge, Mo, Nb, P, Re, S, Si, Ta, Ti, W and Zr multi-element standard (MES 5), 10 µg/ml, in H_2_O tr. HF/tr. HNO_3_ (e.g. TruQ ms Multi-Element Calibration Standard 5, from PerkinElmer LAS GmbH, 63110 Rodgau, Germany, Art. No.: N9300235)
- Al, Ag, As, B, Ba, Be, Bi, Ca, Cd, Cs, Co, Cr, Cu, Fe, In, K, Li, Mg, Mn, Mo, Na, Ni, Nb, Pb, Rb, Sb, Se, Sr, Ti, Tl, V, U and Zn multi-element quality control standard (QC33), 100 mg/l, in 3% HNO_3_ (e.g. ARISTAR, traceable to NIST SRMs, from VWR International GmbH, 64295 Darmstadt, Germany)
- Ultrapure water (ρ ≥ 18.2 MΩ × cm at 25 °C)
- Argon 5.0, 99.999%
- Helium 6.0, 99.9999%

*Note:*

*The internal standard for this method is lutetium. An internal standard that contains several elements (lutetium, scandium, tellurium and yttrium) was used during the development of the method. This internal standard is suitable for the routine analysis of different kinds of samples. Scandium, tellurium and yttrium are not required for the determination of gadolinium concentrations, but do not negatively affect the measurement results for gadolinium.*

### 4.3 Solutions

The following solutions were prepared using the chemicals listed in Section 4.2:

**Acid digestion mixture: **(65% HNO_3_ , 25% HCl, 2 : 1 (v/v))

Two parts by volume of nitric acid (65%) and 1 part by volume of hydrochloric acid (25%) are carefully mixed in a 500-ml PFA container. To prepare the 25% hydrochloric acid formulation, 185 ml of ultrapure water are placed into a 1-l PFA container and brought to volume with 815 ml HCl (30%).

**Dilution solution: **(0.67–0.69% HNO_3_ in water): for the dilution and stabilisation of samples and standards

1.5 l of ultrapure water are placed into a 2-l PFA container, 20 ml of 67 to 69% nitric acid are added and the container is filled to 2 l with ultrapure water.

**Mobile phase: **(0.67–0.69% HNO_3_ in water): for the quadrupole ICP mass spectrometer

400 ml of ultrapure water are placed into a 500-ml PFA container, 5 ml of 67 to 69% nitric acid are added and the container is filled to 500 ml with ultrapure water.

**Rinsing solution: **(0.65% HNO_3_ in water): for the autosampler/tubes of the quadrupole ICP mass spectrometer

4 l of ultrapure water are placed into a 5-l PP container. 50 ml of 65% nitric acid are added and the container is filled to 5 l with ultrapure water.

**Intermediate dilutions single-element standard gadolinium:** (1-Gd: 100 µg/l and 2-Gd: 1 µg/l)

Stock solution 1-Gd is prepared by pipetting 100 µl of the gadolinium standard for ICP (10 mg/l) into a 15-ml PP vial. The vial is filled to 10 ml with dilution solution using a dispenser and homogenised by shaking. 

Stock solution 2-Gd is prepared by pipetting 100 µl of stock solution 1-Gd into 10 ml of dilution solution and then shaking.

Two batches of stock solution 1-Gd and 2-Gd are prepared using different gadolinium standards for ICP (see Section 4.2).

### 4.4 Calibration and control standards

**Internal standard solution:** (0.2 mg Lu/l in water) 

Internal standards are chosen that do not contain a constituent element of the samples. 

The internal standard solution is prepared by pipetting 0.1 ml of the lutetium standard for ICP (1000 mg/l) into a 500-ml volumetric flask made of PTFE. The flask is filled to the mark with dilution solution (see Section 4.3) and shaken. If the internal standard contains scandium, tellurium and yttrium, then 0.4 ml of the Sc standard (0.8 mg/l), 0.75 ml of the Te standard (1.5 mg/l) and 0.2 ml of the Y standard (0.4 mg/l) are used to prepare the internal standard solution.

All solutions, including the calibration standards and the quality control and sample solutions, are spiked with 0.1 ml of the internal standard solution (to 10 ml). This is equivalent to a lutetium concentration of 2 µg/l.

**Calibration standards:** The calibration standards are prepared by diluting stock solution 1-Gd and 2-Gd (see Section 4.3). For this purpose, the stock solutions in the volumes given in Table 1 and 100 µl of the internal standard solution are placed into vials and filled to a total volume of 10 ml with dilution solution using dispensers and piston pipettes.

**Tab.1:** Preparation of calibration standards

Calibration standard	Volume of 1-Gd [µl]	Volume of 2-Gd [µl]	Internal standard solution [µl]	Concentration [µg Gd/l]
1	-	100	100	0.01
2	-	500	100	0.05
3	10	-	100	0.1
4	50	-	100	0.5
5	100	-	100	1
6	500	-	100	5
7	1000	-	100	10

*Note:*

*During the development of the method, stock solutions 1-Gd and 2-Gd were pipetted into multi-element standards (MES 4 and MES 5) to ensure that the calibration is suitable for the routine analysis of different kinds of samples (see* Section 4.2 *).*

**Control standards:**

The second gadolinium standard solution is used to perform quality control procedures during the analytical runs (gadolinium standard for ICP, 10 mg/l, see Section 4.2). The aim is to verify the accuracy of the calibration standards and the stability over time every working day and over the entire measurement period.

First, stock solution QC33 with an element concentration of 100 µg/l is prepared by diluting the multi-element quality control standard (QC33, 100 mg/l, see Section 4.2). For this purpose, 10 µl of the standard are placed into a 15-ml screw-cap vial (see Section 4.1) and mixed with 9.99 ml of ultrapure water (see Section 4.2).

The quality control solutions are prepared according to the pipetting scheme given in Table 2. After adding 100 µl of internal standard solution (Section 4.4) to each solution, the vials are filled to 10 ml with dilution solution (Section 4.3) using a dispenser. The second batch of stock solution 1-Gd that was not used to prepare the calibration solutions is used for this purpose (Section 4.2).

**Tab.2:** Preparation of the quality control standards

Control standard	Volume of stock solution QC33 [µl]	Volume of 1-Gd [µl]	Internal standard solution [µl]	Concentration [µg Gd/l]
1	10	10	100	0.1
2	100	100	100	1

## 5 Sampling and sample preparation

### 5.1 Sampling

Samples are collected using stationary or personal sampling procedures. The samples taken by personal sampling are collected within the breathing zone. The inlet of the sampling head must remain unobstructed during sampling. Binding assessment criteria have not been established for gadolinium. However, for a full evaluation of the workplace, samples of the metals should be taken both in the inhalable and respirable fractions.

A membrane filter (nitrocellulose) is inserted into the sampling head for respirable dust (FSP) (or into the head for inhalable dust (GSP)) and a pump is connected. The flow-regulated pump draws a sample of air through the membrane filter at a flow rate of 10 l/min. A sampling time of at least two hours is recommended (AGS 2023). If necessary, the sampling time can be extended up to 8 hours. Over a sampling period of 2 hours, this is equivalent to an air sample volume of 1.2 m^3^. A sampling record is kept of the parameters required for determining the concentration in air (air sample volume, temperature, air pressure and relative humidity).

The flow rate must be checked for constancy after sampling. If the deviation from the adjusted flow rate is larger than ± 5%, the sample should be taken again (DIN 2023 b). The loaded sample carrier is removed from the sampling head, a cap is put on the carrier, and the carriers are transported to the laboratory for analysis with as little jarring as possible.

### 5.2 Sample preparation

The samples are digested according to the procedures outlined in the IFA Folder of the German Institute of Occupational Safety and Health with the code number 6015 (IFA-Arbeitsmappe, Kennzahl 6015) (Pitzke et al. 2018) and in Pitzke et al. (2020). 

**Open hot-block digestion:**

The loaded filter is transferred to a 25-ml quartz glass digestion vessel using ceramic tweezers and mixed with 10 ml of the acid digestion mixture (see Section 4.3). Glass rods are added to the digestion vessels and air coolers are placed onto the vessels. The vessels are placed into a heating block and heated for two hours to boiling (block temperature approx. 135 °C). After cooling, 10 ml of ultrapure water (ρ ≥ 18.2 MΩ × cm at 25 °C) are carefully added through the air cooler. The mixture is again heated to boiling. After cooling the digested sample, the air cooler and glass rods are removed, the digestion vessel is sealed with a PE stopper and ultrapure water is added to bring the digestion solution to a total volume of 20 ml. The solution is then prepared for ICP analysis.

**Microwave-assisted pressure digestion:**

The loaded filters are transferred to quartz glass digestion vessels that are suitable for microwave-assisted pressure digestion using ceramic tweezers. 10 ml of 65% nitric acid (see Section 4.2) is added. The vessels are sealed with quartz caps and heated for 60 minutes to 240 °C (pressure approx. 60 bar). After cooling, the digestion solution is carefully transferred to a 25-ml quartz glass digestion vessel and filled to 20 ml with ultrapure water (ρ ≥ 18.2 MΩ × cm at 25 °C). The solution is then prepared for ICP analysis.

Dilutions of the samples are prepared for quantitative analysis by placing aliquots of the digested samples into suitable graduated 15-ml PP vials. A dispenser system is used to add 100 µl of the internal standard solution and to fill the vials to a total volume of 10 ml with dilution solution.

A blank value is determined for each series of samples by carrying out all sample preparation and analysis steps (open hot-block or microwave-assisted pressure digestion) with at least two empty filters from the same batch.

## 6 Operating conditions

**Table:** 

**Apparatus:**	Quadrupole ICP mass spectrometer, NexION 2000B, PerkinElmer LAS GmbH, Rodgau, Germany	
**Plasma parameters:**	Optimised for robust plasma conditions and samples/matrices of largely unknown composition (CeO/Ce ≤ 1.5%; Ce++/Ce ≤ 1.5%) RF power: 1550 W	
**Nebuliser:**	MEINHARD nebuliser, concentric, glass	
**Nebuliser chamber:**	Cyclonic C3 high sensitivity, glass, Peltier cooled, with inner tube	
**Injector:**	Quartz, inner diameter 2.0 mm	
**Flow rate:**	Sample solution: 0.4 ml/min	
	Carrier gas: 1.0 l/min	
**Detector:**	Secondary electron multiplier (SEM); two-stage detector “Dual Stage”	
**Isotope:**	Gadolinium 158 amu or 157 amu	
**Measurement modes:**	Standard, kinetic energy discrimination (KED)	
**Interference minimisation:**	Isobaric:	A correction equation is used to minimise interference caused by the major interferent ^163^Dy in standard mode.
	Correction of interference:	Isobaric interference by ^163^Dy is corrected by “–0.004016 × ^163^Dy”.
	Polyatomic:	KED mode to remove the interferent ArCl and at the same time verify the measurement in standard mode
**Internal standard, isotope:**	Lutetium 175 amu	
**Measurement solutions:**	An aliquot of the sample solution is diluted to a ratio of at least 1 : 10 (v/v). The internal standard solution is added using the same procedure as for the calibration standards (see Section 4.4). If the measurement results lie outside of the linear range of the calibration function, further dilutions, e.g. 1 : 100 and 1 : 1000 (v/v), must be prepared.	

All solutions must be freshly prepared every working day, particularly the calibration solutions and the quality control samples (see Section 4.4).

## 7 Analytical determination

After preparation, the diluted sample is injected into the ICP mass spectrometer using an autosampler and analysed under the operating conditions described in Section 6.

*Note:*

*All solutions must be freshly prepared every working day, particularly the calibration standards and the quality control samples.*

## 8 Calibration

The calibration standards described in Section 4.4 are used to obtain the calibration functions.

The calibration standards are injected into the ICP mass spectrometer and analysed according to the same procedure as the sample solutions. The quotients obtained by dividing the gadolinium concentrations of the calibration standard by the internal standard lutetium are plotted against the intensities of the measured pulses. The calibration function is linear over the examined concentration range.

To check the calibration function, control samples must be analysed at the beginning and at the end of each series. The calibration must be performed every working day.

## 9 Calculation of the analytical result

The metal concentration in the workplace air is calculated based on the concentration of gadolinium in the measurement solution that was determined by a data analysis programme. The results obtained by the programme are based on the calculated calibration function. The concentration of gadolinium in the workplace air is calculated (using the same procedure as for other metals) from the gadolinium concentrations taking the respective dilutions and the air sample volume into account. Equation 1 is used to calculate the concentration of analyte in the air:


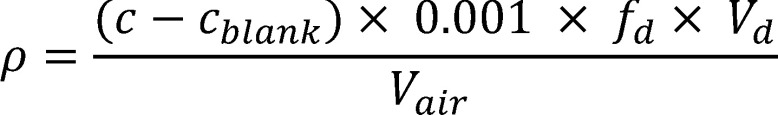
(1)

where: 

**Table:** 

*ρ*	is the mass concentration of the substance in the air sample in mg/m^3^
*c*	is the concentration of gadolinium in the measurement solution in µg/l
*c_blank_*	is the concentration of the blank value (mean) in µg/l
*0.001*	is the conversion factor [µg → mg]
*f_d_*	is the dilution factor (generally a dilution of 1 : 10)
*V_d_*	is the volume of the sample solution in l
*V_air_*	is the air sample volume in m^3^

## 10 Reliability of the method

The characteristics of the method were determined according to the standards DIN EN 482 (DIN 2021) and DIN EN ISO 21832 (DIN 2020). The limits of detection and quantification were determined according to DIN 32645 (DIN 2008). The software QMSys GUM Professional (Qualisyst n.d.) was used to carry out the uncertainty calculations.

### 10.1 Precision

The coefficient of variation was determined as follows: sets of 8 filters were spiked with 3 different concentrations using the gadolinium standard solution (10 000 mg/l, cf. Section 4.2) and analysed following all steps of the analytical procedure.

20 µl of the gadolinium standard solution (equivalent to 0.167 mg Gd/m^3^) was pipetted onto 8 nitrocellulose filters. To determine the precision for gadolinium concentrations of 1.25 mg/m^3^ and 2.50 mg/m^3^, it was necessary to spike the filters with amounts of 150 µl and 300 µl, respectively. However, as these volumes cannot be applied directly to the filters, the filters were first transferred to digestion vessels and then spiked in the vessels.

After the filters were dried for 24 hours in the laboratory air at room temperature, the filters were digested by open hot-block digestion or by microwave-assisted pressure digestion as outlined in the IFA Folder with the code number 6015 (Pitzke et al. 2018). The digested samples were diluted (dilution factor 100 000) and analysed. The data for precision given in Table 3 were determined from the results.

**Tab.3:** Coefficients of variation (*V_x_*) for gadolinium obtained from spiking experiments with n = 8 determinations per concentration

Volume of standard solution [µl]	Spiked mass [µg]	Concentration^a)^ [mg/m^3^]	*V_x_* open hot-block digestion [%]	*V_x_* microwave-assisted pressure digestion [%]
20	200	0.167	3.5	4.5
150	1500	1.25	3.2	4.0
300	3000	2.50	3.4	4.2

a) The concentration is obtained for a sampling period of 2 hours at a volumetric flow rate of 10 l/min and a digestion volume of 20 ml.

### 10.2 Recovery

As aerosols of varying chemical composition and physical properties are found in different work areas, it is not possible to provide recovery data that are generally valid for the entire procedure. The analytical recovery is defined as 100% for samples prepared as described above in conformity with the standard DIN EN ISO 21832 (restricted to the element Gd and Gd compounds that are soluble in the described system) (DIN 2020).

The following procedure was chosen because the investigated gadolinium concentrations are in the ultratrace range, which makes it difficult to reliably weigh the reference materials. The chosen procedure represents a pragmatic worst-case scenario for the digestion procedure:

The method used for preparation and analysis was verified by means of a certified pure substance (gadolinium 99.9%, see Section 4.2) and a certified metal compound (gadolinium(III) chloride (GdCl_3_ ), anhydrous, see Section 4.2). Gadolinium concentrations of 0.167 mg/m^3^, 1.25 mg/m^3^ and 2.5 mg/m^3^ were included to cover the minimum measurement range. Recovery experiments were also carried out in the range of the determined limit of quantification to cover the limit of quantification and the range of the analytical procedure (see Table 4). In this experiment, not only the recovery, but also the coefficient of variation for each concentration was calculated as a measure of reproducibility.

To enable comparisons, the recovery was determined using both of the digestion procedures described above. The open hot-block digestion procedure described in Section 5.2 was carried out with double the sample size. Approx. 7 mg of the substances (see Table 4) in powder form were weighed onto nitrocellulose filters and another nitrocellulose filter was added to adjust the matrix to the larger sample size. The filters were transferred to 50-ml quartz glass digestion vessels and 20 ml of the acid digestion mixture (see Section 4.3) were added. Glass rods were placed into the vessels, and the samples were then heated for 2 hours to boiling at a block temperature of 135 °C. After cooling to below 100 °C, 20 ml of ultrapure water were added to each of the samples through the air cooler and the samples were heated again for approx. 30 minutes at 135 °C. The solutions prepared as described did not contain any visible particles. The solutions prepared with the gadolinium powder contained gadolinium in concentrations of approx. 170 mg Gd/l and those prepared with the gadolinium(III) chloride powder contained concentrations of 110 mg Gd/l. 

Microwave-assisted pressure digestion was carried out by weighing approx. 7 to 9 mg of the substances listed in Table 4 onto nitrocellulose filters. Ultrapure water was added (see below) to obtain samples double in size and an additional nitrocellulose filter was again used to adjust the matrix. 10 ml of nitric acid (65%) were added to the substances and microwave-assisted pressure digestion was performed under the conditions given in Section 5.2. After cooling, the digested samples were transferred to 50-ml quartz glass vials and 30 ml of ultrapure water were added. The solutions prepared as described did not contain any visible particles. The solutions prepared with the gadolinium powder contained approx. 200 mg Gd/l and those prepared with the gadolinium(III) chloride powder contained 110 mg Gd/l.

The reference solutions that were prepared as described were each used to prepare two intermediate dilutions that contained calculated concentrations of 200 and 10 µg Gd/l. The first intermediate dilution (200 µg/l) was prepared using the stock solutions made with the gadolinium powder by taking 60 µl of the stock solution prepared by open hot-block digestion and 50 µl of that prepared by microwave-assisted pressure digestion and adding dilution solution by dispenser to obtain a total volume of 50 ml. The second intermediate dilution (10 µg/l) was prepared by taking 2500 µl of the first intermediate dilution and diluting to 50 ml final volume. 

The first intermediate dilution (200 µg/l) was prepared using the stock solutions made with the gadolinium(III) chloride powder by taking 95 µl of the stock solution prepared by open hot-block digestion and 200 µl of that prepared by microwave-assisted pressure digestion and adding dilution solution by dispenser to obtain a total volume of 50 ml. To determine the recovery within the minimum measurement range, the first intermediate dilution with a concentration of 200 µg Gd/l was diluted with the dilution solution to obtain sets of eight samples containing gadolinium in concentrations equivalent to concentrations in air of 0.167 mg/m^3^, 1.25 mg/m^3^ or 2.50 mg/m^3^. The samples were then analysed. To determine the recovery in the range of the limit of quantification, the second intermediate dilution with a concentration of 10 µg Gd/l was diluted in the same manner to obtain sets of eight samples containing gadolinium in concentrations equivalent to concentrations in air of 0.002 µg/m^3^ (or 0.001 µg/m^3^ in the case of gadolinium(III) chloride). The samples were then analysed. Additionally, two empty filters (blank filters) were processed through all steps of the analytical procedure. A blank value correction was carried out to calculate the results (see Section 9).

The mean recoveries calculated for the open hot-block digestion procedure by quantitative analysis were 95.1% for gadolinium and 95.3% for gadolinium obtained from gadolinium(III) chloride. The mean recoveries calculated for the microwave-assisted pressure digestion procedure were 94.7% for gadolinium and 93.5% for gadolinium obtained from gadolinium(III) chloride.

**Tab.4:** The recovery (*η*) and coefficient of variation (*V_x_*) for open hot-block digestion and microwave-assisted pressure digestion for n = 8 determinations per concentration

Tested substance	Concentration of the intermediate dilution used	Mass of Gd loaded onto the filter^a)^	Gd concentration^b)^	Open hot-block digestion		Microwave-assisted pressure digestion	
				***η***	*V_x_*	*η*	*V_x_*
	[µg Gd/l]	[µg]	[mg/m^3^]	[%]	[%]	[%]	[%]
Gd	10	0.0011	9.02 × 10^–7^	96.4	1.4	95.5	0.95
Gd	200	200	0.167	95.4	1.9	95.1	3.2
Gd	200	1500	1.25	94.4	1.5	94.5	0.57
Gd	200	3000	2.50	94.4	0.70	93.8	1.5
GdCl_3_	10	0.0011	9.02 × 10^–7^	96.1	1.5	95.4	1.8
GdCl_3_	200	200	0.167	95.0	2.8	92.3	1.2
GdCl_3_	200	1500	1.25	95.3	1.2	93.5	1.1
GdCl_3_	200	3000	2.50	94.6	0.82	92.9	0.94

a) The mass was not determined directly by weighing, but by reverse calculation after factoring in the dilution steps and the digestion volume.

b) The concentration is obtained for a sampling period of 2 hours at a volumetric flow rate of 10 l/min and a digestion volume of 40 ml.

### 10.3 Expanded uncertainty

The expanded uncertainty was determined by estimating all relevant influencing parameters. There are two main sources of uncertainty in the measurement results: the estimated uncertainty arising from the sampling procedure and that arising from the analytical procedure.

The uncertainties of the method were estimated by determining the uncertainties associated with the air sample volume and the sampling effectiveness for respirable dusts (DIN 2020). However, if this method is used to determine the concentration of gadolinium in the inhalable dust fraction, the uncertainty analysis must take into account the uncertainties that are relevant for sampling the inhalable dust fraction, which differ from those of the respirable fraction (DIN 2020). In this case, the calculated uncertainty represents the worst-case scenario. When sampling the respirable fraction, there are more contributions to uncertainty that must be taken into consideration than for the inhalable fraction. As a result, the expanded uncertainty for the inhalable fraction is expected to be lower.

Uncertainties arising from analysis may occur at any point of the analytical procedure including digestion, dilution, calibration, recovery and precision. The analytical uncertainties for this method are dependent on the concentration and were determined using the QMSys GUM Professional software (Qualisyst n.d.). The data are shown in Table 5.

The combined uncertainty for the entire method was calculated by combining the contributions from all sources of uncertainty. These values are multiplied by the expansion factor k = 2 to obtain the percentage for the expanded uncertainty (*U*) for the entire method (see Table 5) for the tested concentrations of gadolinium at a volumetric flow rate of 10 l/min and a sampling period of two hours.

**Tab.5:** Expanded uncertainty (*U*)

Tested substance	Concentration [mg/m³]^a)^	*U* [%]
Gd	0.167	25.0
Gd	1.25	23.6
Gd	2.50	23.5

a) The concentration is obtained for a sampling period of 2 hours at a volumetric flow rate of 10 l/min and a digestion volume of 40 ml.

### 10.4 Influence of humidity

If the relative humidity exceeds 50% and if the substances occur in the work area in dissolved form, the filter cassette should be loaded with two quartz fibre filters as sample carriers.

### 10.5 Limit of quantification

As the method was being developed, the limit of quantification was calculated using the equation given in the standard DIN 32645 (DIN 2008) by applying the blank value method.

For this purpose, sets of ten empty filters were processed through all preparation and analysis steps of the open hot-block digestion procedure and the microwave-assisted pressure digestion procedure (see Section 5.2). After analysis, the standard deviation of the signal and the mean signal value, the analyte blank values for all laboratory blank samples obtained from the filters, reagents and vessels used, and the analytical limit of quantification *c_LOQ_* were determined as specified in DIN 32645 (see Equation 2). The calculation can be performed only if the slope *b* of the calibration function is known.


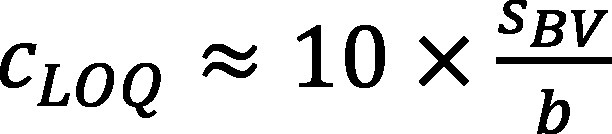
(2)

where:

**Table:** 

*c_LOQ_*	is the limit of quantification in µg/l
*s_BV_*	is the standard deviation for replicate determinations of the blank value
*b*	is the slope of the calibration function, which is also used during routine operations

The relative limits of quantification of the method were determined using Equation 3. The values are shown in Table 6.


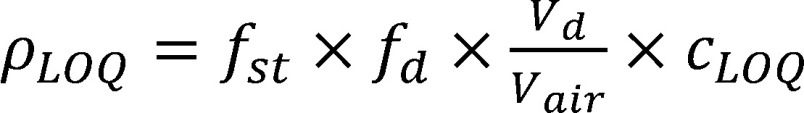
(3)

where:

**Table:** 

*ρ_LOQ_*	is the limit of quantification of the substance in air in mg/m^3^
*f_st_*	is the conversion factor (stoichiometric factor)
*f_d_*	is the dilution factor (generally 1 : 10)
*V_d_*	is the volume of the sample solution
*V_air_*	is the air sample volume in m^3^

**Tab.6:** Limits of quantification for gadolinium (k = 3)

Type of digestion	Absolute limit of quantification [µg/l]	Relative limit of quantification^a)^ [µg/m^3^]
Open hot-block digestion	0.013	0.002
Microwave-assisted pressure digestion	0.005	0.001

a) The limit of quantification was obtained for a sampling period of 2 hours at a volumetric flow rate of 10 l/min and a digestion volume of 20 ml.

The instrument limit of detection (IDL) was determined by analysing twelve calibration blank solutions. For this purpose, twelve samples were prepared with 9.90 ml of dilution solution and 0.10 ml of the internal standard solution. The IDL is the concentration required to produce a signal equivalent to three times the standard deviation of the measurements by ICP-MS and was calculated to be 2.6 × 10^–5^ µg/m^3^ for gadolinium.

### 10.6 Storage stability

The storage stability of the loaded sample carriers was analysed by spiking the filters with concentrations equivalent to one tenth and twice the general limit value for dust (R dust) and with a concentration close to the limit of quantification.

Three sample carriers per concentration and storage day were spiked with different volumes (see Table 7) of the gadolinium standard for ICP (c = 10 000 mg/l; see Section 4.2) to cover the lower and the upper concentration of the analytical measurement range. To analyse a concentration close to the limit of quantification, a dilution was prepared with the gadolinium standard for ICP (c = 10 mg/l; see Section 4.2) by placing 100 µl of the solution into a vial, adding dilution solution (see Section 4.3) to a total volume of 10 ml and then shaking. This dilution (c = 0.100 mg/l) was used to spike sets of three sample carriers (see Table 7). All filters were dried at room temperature and then stored in a fume hood. The filters were prepared and analysed on two measurement days per week. The day of spiking is regarded as storage day 0.

Filters spiked with gadolinium in concentrations ranging from 0.167 mg/m^3^ to 2.5 mg/m^3^ are stable for up to four weeks. However, exceeding a storage period of 14 days is not recommended because solutions with concentrations close to the limit of quantification are guaranteed to be stable only during this period of time.

**Tab.7:** Comparative measurements for the storage of filters spiked with gadolinium in concentrations 0.1 and 2 times the general limit value for dust (R fraction) and in a concentration close to the limit of quantification

Storage period [days]	Concentration of the spiking solution [mg/l]	Volume of the spiking solution [µl]	Concentration^a)^ [mg/m^3^]	Mean recovery [%]
31	10 000	20	0.167	100.0
31	10 000	300	2.50	101.5
14	0.100	100	0.000002	92.43

a) The limit of quantification is obtained for a sampling period of 2 hours at a volumetric flow rate of 10 l/min and a digestion volume of 20 ml.

### 10.7 Selectivity

Isobaric interferences must be avoided, if possible, by choosing other isotopes. Polyatomic interferences can largely be reduced by establishing robust plasma conditions (oxide rate and rate of doubly-charged ions ≤ 2%) as well as by applying the described collision cell technology followed by kinetic energy discrimination.

In samples of mainly unknown composition, interference may still occur due to high concentrations of other elements and compounds. Therefore, the results of the analysis must generally be checked for possible interference (e.g. by determining several isotopes and modes). If necessary, a suitable dilution must be chosen to achieve valid results. 

The presence of ^163^Dy is one source of interference with gadolinium and its compounds (isobaric interference). This interference can be minimised by applying the interference correction equation included in the data analysis software.

Furthermore, interferences with gadolinium caused by high concentrations of ^150^Sm^++^, ^150^Nd^++^ and ^153^Eu^++^ ions must be taken into consideration. Isotopes of these metals should be included in the analysis at least as monitor ions.

Interference with gadolinium by ArCl may occur during the analysis in standard mode; therefore, the Gd concentration that is obtained should additionally be verified in KED mode.

## 11 Discussion

The analytical method provides a means of determining gadolinium and its compounds in the workplace air in concentrations ranging from 0.1 to 2 times the general limit value for dust (respirable fraction) of 1.25 mg/m^3^. Assessment criteria have not been established for gadolinium. Therefore, the minimum measurement range is relatively large with values between 0.125 and 2.50 mg/m^3^. As gadolinium generally occurs at the workplace in low concentrations, the calibration was adjusted for a lower concentration range (from 0.01 µg/l or 0.002 µg/m³ to 10 µg/l or 1.67 µg/m^3^). The reliability of the results obtained by this method for lower concentrations was verified by recovery and storage experiments performed at the limit of quantification. If necessary, samples with higher concentrations must be diluted to this concentration range. Once assessment criteria have been established for gadolinium, a new minimum measurement range must be determined and the recovery data must be revalidated to verify the analytical method.

The ^157^Gd isotope and the ^158^Gd isotope may both be used. These isotopes yield similar results, irrespective of whether the analysis is carried out in standard or collision mode.

In general, all conditions must be adapted to the ICP-MS device that is used, particularly those relating to the analysis of the samples.

During the method verification process, additional validation experiments were carried out with the complex compound gadobutrol [770691-21-9], which is used as a contrast agent in MRI. Similar results were obtained for this compound using this method. If the method is used for the analysis of other gadolinium compounds, it must first be verified for these compounds.
